# Study Protocol for a randomized controlled trial of mentalization based therapy against specialist supportive clinical management in patients with both eating disorders and symptoms of borderline personality disorder

**DOI:** 10.1186/1471-244X-14-51

**Published:** 2014-02-21

**Authors:** Paul Robinson, Barbara Barrett, Anthony Bateman, Az Hakeem, Jennifer Hellier, Fenella Lemonsky, Clare Rutterford, Ulrike Schmidt, Peter Fonagy

**Affiliations:** 1 Division of Psychiatry, University College London, Gower Street, London WC1E 6BT, UK; 2 Research and Development Department, Barnet Enfield and Haringey Mental Health Trust, St Ann’s Hospital, St Ann’s Road, London N15 3TH, UK; 3 Department of Health Economics, Institute of Psychiatry, de Crespigny Park, London SE5 8AF, UK; 4 Halliwick Centre, St Ann’s Hospital, St Ann’s Road, London N15 3TH, UK; 5 The Dartmouth Park Unit, London N19 5NX, UK; 6 Department of Biostatistics, King's College Clinical Trials Unit, Institute of Psychiatry, King's College London, London SE5 8AF, UK; 7 Expert by Experience, London, UK; 8 King's College Clinical Trials Unit, Institute of Psychiatry, King's College London, London SE5 8AF, UK; 9 Section of Eating Disorders, Institute of Psychiatry, de Crespigny Park, London SE5 8AF, UK; 10 Psychology Department, University College London, Gower Street, London WC1E 6BT, UK

**Keywords:** Borderline, Anorexia, Bulimia, Mentalization, Self-harm, Psychotherapy, Attachment, Personality, RCT, Eating

## Abstract

**Background:**

The NOURISHED study: Nice OUtcomes for Referrals with Impulsivity, Self Harm and Eating Disorders.

Eating Disorders (ED) and Borderline Personality Disorder (BPD) are both difficult to treat and the combination presents particular challenges. Both are associated with vulnerability to loss of mentalization (awareness of one’s own and others’ emotional state). In BPD, Mentalization Based therapy (MBT) has been found effective in reducing symptoms. In this trial we investigate the effectiveness and cost-effectiveness of MBT adapted for Eating disorders (Mentalization Based Therapy for Eating Disorders (MBT-ED)) compared to a standard comparison treatment, Specialist Supportive Clinical Management (SSCM-ED) in patients with a combination of an Eating Disorder and either a diagnosis of BPD or a history of self-harm and impulsivity in the previous 12 months.

**Methods/Design:**

We will complete a multi-site single-blind randomized controlled trial (RCT) of MBT-ED vs SSCM-ED. Participants will be recruited from three Eating Disorder Services and two Borderline Personality Disorder Services in London. Participants allocated to MBT-ED will receive one year of weekly group and individual therapy and participants allocated to SSCM-ED will receive 20 sessions of individual therapy over 1 year. In addition, participants in both groups will have access to up to 5 hours of dietetic advice. The primary outcome measure is the global score on the Eating Disorders Examination. Secondary outcome measures include total score on the Zanarini BPD scale, the Object Relations Inventory, the Depression Anxiety Stress Scales, quality of life and cost-effectiveness. Measures are taken at recruitment and at 6 month intervals up to 18 months.

**Discussion:**

This is the first Randomised Controlled Trial of MBT-ED in patients with eating disorders and symptoms of BPD and will provide evidence to inform therapy decisions in this group of patients. During MBT-ED mentalization is encouraged, while in SSCM-ED it is not overtly addressed. This study will help elucidate mechanisms of change in the two therapies and analysis of therapy and interview transcripts will provide qualitative information about the conduct of therapy and changes in mentalization and object relations.

**Trial registration:**

ISRCTN51304415

## Background

Patients with eating disorders (ED) combined with symptoms of borderline personality disorder (BPD) present particular therapeutic challenges. Symptoms include weight loss, bulimia, self harm, emotional lability and impulsive behaviours. During treatment, while some symptoms may improve, others can worsen. Therefore treatment needs to address both the eating and personality disorders, and individuals with both conditions are more likely than those with eating disorders alone to have a complicated course with atypical disorders and migration from one disorder to another [1]. Borderline Personality Disorder has been shown to respond to Mentalization Based Therapy (MBT) [2] which targets vulnerability to loss of self-reflectiveness (mentalizing) which is thought to occur in both BPD [3,4] and Eating Disorders [5,6]. This project will test whether MBT is an effective treatment for this troubled group of patients.

Eating disorders are among the most common causes of ill health in young people, affecting some 3-4% of women and 0.3% of men [7,8]. The morbidity associated with eating disorders is of several types. Low weight, electrolyte imbalance and nutrient deficiency leads to serious physical problems which can be fatal if extreme. Other problems include depression and anxiety [9], social isolation, family, relationship and occupational difficulties and a substantially reduced quality of life [10]. BPD has a prevalence of 0.7% but a roughly equal sex distribution [11,12]. It has a broad impact on psychological, family and social functioning [13]. Symptoms include mood fluctuations, including depression, outbursts of anger, deliberate self harm, suicidal behaviour and disturbed relationships.

The two sets of conditions occur together more commonly than would be expected. The combination of Eating and Borderline Personality Disorder symptoms was noted by Lacey [14] who found that 80% of a series of patients with bulimia nervosa engaged in at least three self damaging behaviours and coined the term “Multi-impulsive bulimia”. The proposed trial will study this group, although the criteria and the name given, (ED with symptoms of BPD), differ. Lacey [14] and Zeeck et al [15] pointed out their very severe morbidity and the difficulties in treatment they present. The direct and indirect costs of these problems have not been reported, although the costs of ED [16-18] and BPD [19] are both high. Moreover, there is some evidence [14,20] that engagement in treatment of patients suffering from both disorders is poor.

It has been shown that nearly 30% of ED patients meet criteria for any personality disorder, while 6.2% have BPD [21]. In the present study, patients with ED complicated by some of the most damaging BPD symptoms, namely suicidal behaviour, deliberate self harm and impulsive behaviours (although not always fulfilling all BPD criteria) will be included. A review of recent patients treated at the Eating Disorders Unit at the main study site, St Ann’s Hospital, suggested that some 20% of patients seen fulfilled study criteria. Treatment of eating disorders has a limited evidence base: Cognitive Behaviour Therapy (CBT) for bulimia nervosa and binge eating disorder is the only treatment for which NICE has cited class A Randomised Controlled Trial (RCT) evidence [18]. For Borderline Personality Disorder, Mentalization Based Therapy [22], Dialectical Behaviour Therapy (DBT) [23] and Cognitive Behaviour Therapy [24] have shown benefit in RCTs. NICE [25] calls for more Randomised Trials to evaluate all these and other therapies. For patients with both ED and BPD, DBT has shown promise in small pilot studies [26,27]. MBT has a theoretical basis in attachment theory and deficits in primary attachments have been shown in both Eating Disorders [28] and Borderline Personality Disorder [29]. Skårderud [30,31] has argued for the importance of impaired mentalization and the relevance of MBT in in anorexia nervosa. MBT therefore has a theoretical basis which may have significance for the aetiology as well as the treatment of ED/BPD. We have therefore used MBT in the current trial, adapted for participants with Eating Disorders and the modified therapy is called MBT-ED [32].

Specialist Supportive Clinical Management (SSCM) was developed as a control therapy in trials of treatment for anorexia nervosa [33]. We chose it as a control treatment because it is a manualized treatment that approximates to treatment as usual for eating disorders. In the current trial patients can have any eating disorder diagnosis, and so the Therapy manual for SSCM was adapted for use with any eating disorder and the modified therapy called SSCM-ED. Except for the paragraph headed “Control condition” below, we have used the terms MBT and SSCM in this paper to indicate the modified therapies used in the study.

The intended participants in this trial suffer from complex problems which can adversely influence clinical care. We therefore embarked on a randomized controlled trial of Mentalization Based Therapy against Specialist Supportive Clinical Management in patients with a diagnosis of an eating disorder and either BPD or symptoms of BPD and here present the protocol for that study.

### Objectives of the trial

#### Primary objective

To ascertain whether Mentalization Based Therapy is clinically effective at reducing observer rated symptoms of Eating Disorder, using an accepted measure, the Eating Disorders Examination, in patients with combined eating and borderline personality disorder symptoms up to 18 months post randomisation compared to Specialist Supportive Clinical Management.

#### Secondary objectives

1. To ascertain whether Mentalization Based Therapy is cost effective at reducing observer rated symptoms of Eating Disorder, using an accepted measure, the Eating Disorders Examination, in patients with combined eating and borderline personality disorder symptoms up to 18 months post randomisation compared to Specialist Supportive Clinical Management.

2. To determine whether Mentalization Based Therapy is clinically effective at reducing symptoms of Borderline Personality Disorder as measured by the Zanarini-BPD scale, an accepted measure of BPD symptoms [34] in patients with combined eating disorders and borderline personality disorder symptoms up to 18 months post randomisation compared to Specialist Supportive Clinical Management.

3. To ascertain whether adverse events in the two groups differ.

4. To study possible mediators of change (e.g. reflective function, mentalizing, object relations, personality) in order to provide information on how changes might occur during therapy.

5. To ascertain whether MBT is a cost-effective treatment for BPD compared to SSCM.

## Methods

### Study design

This is a multi-centre study across five Clinical Centres, three NHS Eating Disorder Units and two NHS Personality Disorder Units in three different NHS Trusts.

The design is a single-blind (researchers being blind) randomised controlled trial of Mentalization Based Therapy for eating disorders (MBT-ED), with an intensity of one individual and one group session per week for one year, compared to Specialist Supportive Clinical Management adapted for all eating disorders (SSCM-ED), a standard treatment for eating disorders, which comprises one session every 1 to 4 weeks for 20 to 26 sessions All trial participants have access to 5 hours of dietetic advice over the course of treatment.

Participants will be recruited from the participating clinics and will include both new referrals and patients currently receiving care. Randomisation will be remote so ensure researcher blindness to treatment allocation.

Treatment will begin following randomization for 12 months (MBT-ED group) or for up to 12 months (SSCM-ED group). All participants will be asked to complete assessment questionnaires and to participate in assessment interviews at 6, 12 and 18 months follow-up.

### Participants

Inclusion criteria

1. Over 18

2. Eating Disorder diagnosis (DSM IV [35])

3. BPD symptoms

4. Able and willing to provide written informed consent

The criteria for “BPD symptoms” are that the patient fulfils both behavioural criteria of the DSM IV, namely 1. impulsivity in at least two areas that are potentially self-damaging (e.g. spending, sexual behaviour, substance abuse, reckless driving, binge eating). 2. recurrent suicidal behaviour, or self-mutilating behaviour.

Exclusion criteria

1. Current psychosis based on the MINI examination [36]

2. Current inpatient or day-patient (attending 3 or more days per week)

3. Currently in individual or group psychological therapy

4. Received MBT <6 months prior to randomization

5. Organic brain disease leading to significant cognitive impairment

6. Body Mass Index (BMI) less than 15 kg/m^2^ (Normal range 19-25, Anorexia Nervosa <17.5)

### Design and procedure

#### Recruitment and consent

We aim to include all patients with current eating disorders and Borderline Personality Disorder symptoms as defined above. Participants may be underweight, normal weight or overweight, although because of the increased risk of physical decompensation those severely malnourished (with a BMI of under 15) are excluded. We will therefore be assessing the effectiveness of MBT in patients with different eating and weight problems. In addition to their eating disorder, we anticipate that most participants will have current self harm and impulsive behaviours and a sub-group will fulfill all criteria for BPD. In addition, a group of participants will fulfill criteria for BPD without necessarily experiencing self harm or impulsivity.

The initial introduction to the study will come from the treating clinician. If interested in obtaining more information, the clinician will provide the Participant Information Sheets and Carer Information Sheets. At least 24 hours later, the potential participant will be approached by a member of the research team and if still interested, an interview time arranged. At that meeting the study will be described, outstanding questions answered and the potential participant asked to sign the consent form which will be countersigned by the member of the research team.

The participant will then receive the following interviews:

Eating Disorders Examination (EDE) [37,38]

Reflective Function Questionnaire (RFQ)^a^

Reading the mind in the eyes test [39].

Object Relations Inventory (ORI) [40].

Acts of Deliberate Self Harm Inventory (ADSHI) [41].

MINI International Neuropsychiatric Schedule (MINI), [36].

Structured Clinical Interview for DSM-IV Axis II Personality Disorders (SCID-II), [42].

Global Assessment of Functioning (GAF) [35].

Zanarini-BPD (ZAN-BPD) [34].

Euroqol (EQ5D) [43].

Depression, Anxiety, Stress Scales -21 (DASS 21) [44].

Modified Social Adjustment Scale (SAS) [45].

Adult Service Use Schedule (AD-SUS) [46].

Big Five personality Inventory (BFI) [47].

Apart from the MINI and the SCID II which are only administered at baseline for diagnostic purposes, all instruments will be repeated at 6, 12 and 18 months post randomization.

The original signed consent form will be retained in the Investigator Site File, with a copy in the participants’ hospital medical notes, and a copy retained by the participant for their information. The participant will specifically consent to their General Practitioner (GP) being informed of their participation in the study.

The right to refuse to participate without giving reasons will be respected.

#### Trial monitoring safety assurance

Three trial monitoring committees will be formed:

1. Trial Management Committee (TSC): This will manage the day to day running of the trial and comprise the Chief Investigator, the Trial Manager and relevant members of the research team, as appropriate. It will meet weekly.

2. Independent Data Monitoring Committee (iDMC): This will have an open and a closed session at each meeting. The chair will be an independent academic and members will include an independent Eating Disorders expert and an Independent general Academic Psychiatry expert. The Chief Investigator and the Trial Statistician, who will provide reports, the Trial Manager and the Trial Statistician will be in attendance. Of the latter, only the Trial Statistician will attend the closed part of the meeting. In the closed part, summaries of follow-up assessments and adverse events, will be available for each of the treatment groups (labelled a and b). iDMC meetings will be held annually, or more often if required and the chair will advise the Trial Steering Committee of their recommendations regarding continuation, stopping early or modifications to the protocol.

3. Trial Steering Committee (TSC): The TSC will be chaired by an independent expert and members will include experts in Eating Disorders and General Psychiatry (different from those in the iDMC) as well as user and carer representatives, a lead trust representative and the Chief Investigator, who will provide a report, the Trial Manager and the Trial Statistician. Meetings will be annual, or more frequently should the need arise.

#### Randomisation, allocation concealment and protection against bias

Due to the nature of the intervention blinding of participants and therapists will not be possible. However the Trial Statistician and Research Workers responsible for the collection of the assessments will remain blind to treatment allocation during the trial and primary analyses. Participants will be asked to be careful not to divulge what sort of therapy they have had, or other information that could interfere with the Research Worker’s blindness to treatment group.

Following completion of the baseline questionnaire, patients who consent to take part in the trial are randomly allocated to MBT or SSCM (ratio 1:1) by sites via an online system based at the King’s Clinical Trials Unit based at the Institute of Psychiatry in London. The method of randomisation of participants will be by block randomisation stratified by BMI (15.0-18.5, 18.6-24.9, >25) and service type (eating disorders unit or personality disorders unit). Randomly varying block sizes will be implemented in order to maintain pre-randomisation allocation concealment.

#### Safety monitoring

Safety will be monitored in several ways.

1. Physical safety: Clinical features that influence safety such as weight, muscle strength, serum potassium and electrocardiograph will be identified at initial assessment. Monitoring will be done at intervals recommended by the Centre Clinical Lead. If changes occur, they will be discussed in supervision and if appropriate, with a Clinical Centre doctor. Monitoring will be done at the Clinical Centre, or by the GP. If the latter, agreement in advance will be obtained from the GP to do the monitoring tests. The therapist will be responsible for making sure that safety monitoring is carried out and results obtained and interpreted by an appropriate professional, generally a doctor. Significant results will be communicated to the GP.

2. Self harm and suicide: If a participant discloses thoughts of self harm or suicide during a therapy session, these will be explored in the session. Following the session the findings will be discussed with a supervisor, a doctor, or senior clinical staff at the Clinical Centre and appropriate action decided upon. This could include a session shortly after the current session, either with the therapist or another staff member, a referral for urgent psychiatric assessment or a referral for crisis management.

3. Safety outside clinic hours: Clinic Centre staff will be available during office hours, 9am to 5pm, Monday to Friday. Outside these hours participants who are concerned about their safety for any reason will be asked to contact their GPs or, if urgent, to attend Accident and Emergency, calling an ambulance if necessary.

4. Management of crises: Urges to self harm, actual self harm or suicidal intent: During office hours patients will call their therapy centre and speak to a staff member. If necessary the patient will be seen as soon as possible by a staff member. When the patient is seen, support will be given, necessary medical treatment arranged in consultation with a physician and a risk assessment made to inform a decision on referral on to local crisis services for possible admission either to a psychiatric ward or a crisis facility if available. If admitted, the Therapist will maintain contact and the patient returned to the outpatient care of the therapy team as soon as possible as long as the level of risk is thought to be acceptable by the treating team consultant psychiatrist, the Centre Clinical Lead and the Therapist.

5. Physical symptoms: Weight loss, increasing abnormality of physical tests or any other worrying symptoms, will be discussed with the team physician and a management decision made. This might include increased frequency of monitoring, a physician review and consideration of increased dietetic advice, day hospital attendance or inpatient admission. The latter could be to a general hospital nutrition unit, a general adult psychiatry bed or an eating disorders bed.

#### Interventions

All participants will have an allocated psychiatrist, a key worker/therapist who will also provide individual therapy and a dietician who will provide up to 5 hours of advice over the duration of the trial the timing of which will be agreed between the participant and the therapist. From recruitment, Participants will receive treatment for 12 months (MBT group) or up to 12 months (SSCM group).

#### Experimental condition: mentalization based therapy

This treatment is based on the Intensive Outpatient Therapy model [29]. The Therapist will provide a weekly 50 minute individual MBT session. During therapy the Therapist will be responsible for making sure that physical measures are monitored (e.g. at the clinic, or by the GP), consulting with the clinic physician as required. Participants will also attend weekly group Mentalization Based Therapy for 90 minutes. Therapy will last for 12 months after which patients will be reassessed by a member of the trial clinical team and referred for further management if required. Each MBT group will have a maximum of 10 participants. Participants will be considered compliant of they attend at least 50% of individual and 50% of group sessions offered.

Other treatments: Families and carers will be offered attendance at existing support services as will control families, and attendance will be monitored. Should patients access treatment outside the trial, this will be monitored and specified at the 6 monthly interviews.

#### Control condition **-** specialist supportive clinical management

SSCM was developed by McIntosh et al [33] as a standard treatment for anorexia nervosa (AN) and has been used in several AN trials. [48-50]. It has been modified in collaboration with Dr McIntosh to cover eating disorders in which underweight is not a feature. This extended form has been called SSCM-ED. SSCM has been found to be an effective intervention for anorexia nervosa. It is symptom focused and lacks attention to mentalization or relationships. Hence, it represents a comparison treatment that offers the control participants a proven treatment, which does not aim to alter the specific target symptoms which are addressed in MBT. SSCM offers 20 sessions that can vary in both length (30**-**60 minutes) and frequency (2 per week, to 1 per two weeks depending upon clinical need). At the end of the 20 sessions, therapists can offer a few more sessions if required (up to 6) before ending. Participants will be considered compliant if they attend at least 50% of sessions offered.

### Therapy training, supervision and adherence

#### Mentalization based therapy

Staff providing individual therapy will have a basic clinical training (e.g., Mental health nursing, psychology or social work, art therapy, medicine), will have undergone an introduction to MBT and and treated at least one patient using MBT under appropriate supervision for 6 months. Those providing group therapy will in addition have acted as a co-therapist for 6 months in a supervised MBT group in which the main therapist is an experienced MBT practitioner. Therapists in the MBT arm will not be required to have had specialist training or experience in Eating Disorders.

Supervision sessions will be conducted by trained supervisors, expert in provision and supervision of MBT and with specialist knowledge of Eating Disorders and occur weekly for one hour. Issues arising in both individual and group therapy will be discussed. A proportion of individual sessions will be audio-recorded and the recordings played and discussed in supervision. Participants will sign consent forms for audio recordings.

MBT sessions will be audio recorded and the recordings rated by trained observers to assess the degree to which therapy provided adheres to the treatment model of MBT. The rating scale used will be Bateman and Fonagy [29]. In addition, reports from supervisors on each therapist will assess adherence to model. Therapists found not to reach an accepted level of adherence will be provided with additional training. Group therapy model adherence will be monitored using audio-recordings of therapy sessions and reports from co-therapists.

Patients (accompanied by a relative if the patient wishes) will be offered regular treatment review meetings including medication review, during outpatient sessions in the referring team.

### Specialist Supportive Clinical Management

As is the case for MBT therapists those providing SSCM will have a basic Mental Health Professional training. They will also have had at least 6 months supervised practice with patients with Eating Disorders. In addition, they will be trained in the use of the SSCM-ED manual by a senior clinician over the course of a one-day training. Supervision and discussion of treatment intensity and duration and clinical review will be by a senior clinician in the research team and therapy adherence will be checked during clinical supervision.

Clinical review will be during outpatient sessions in the referring team.

### Assessments, outcome measures, moderators and mediators

Participants will be referred by clinical staff who will be asked to indicate diagnosis and BPD symptoms on a referral sheet. These will be checked at initial research assessment. The EDE will be used to check Eating Disorder diagnoses and the SCID-II [42] and the ZAN-BPD [34] to check Borderline Personality Disorder symptoms and diagnoses.

### Primary outcome measure

The Eating Disorders Examination (EDE) interview [38] will provide information to establish eating disorder diagnosis and severity.

### Secondary outcome measures

The Zanarini-BPD (ZAN_BPD) [34] will measure Borderline Personality Disorder symptoms.

The Acts of Deliberate Self Harm Inventory [24] will be used to monitor self harm.

Health Related Quality of Life will be measured using the EQ5D [43].

Health Economics data (use of health resources) will be monitored using the AD-SUS [46].

Depression, anxiety and stress will be measured using the DASS-21 [44].

### Measures of possible mediators of change

**Figure 1:**
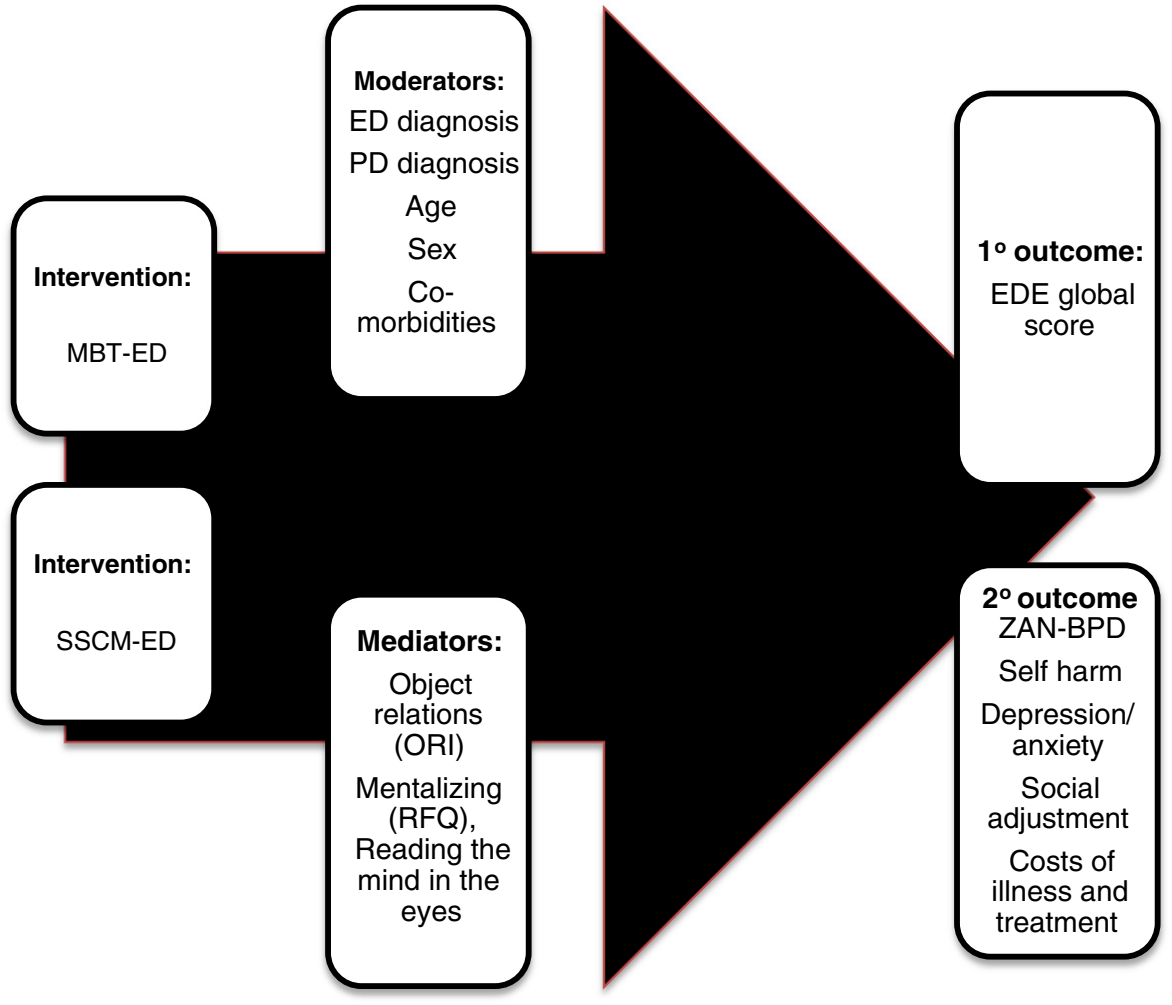
Interventions, mediators, moderators and outcomes studied.

Possible mediators are depicted in Figure 1.

Reflective function will be measured using the Reflective Function Questionnaire (RFQ)^a^.

Mentalization will be assessed using the Reading the Mind in the Eyes Test [39].

The complexity with which participants describe their parents, themselves and their therapists will be assessed using the ORI [40].

Personality will be assessed using the BFI [47].

### Adverse events

Adverse incidents will be reported to the TSC and the iDMC.

### Statistical analysis

#### Power calculation

An overall sample size of 140 (randomised 1:1) would provide over 90% power to detect an effect size of d=1.07 on the Global EDE Score using an analysis of covariance with 2 sided 5% significance tests. This calculation is based on the conservative assumption of a zero correlation between baseline and post treatment scores on the EDE and allows for clustering within therapy groups and 25% attrition. The effect size (1.07) was based on a minimal clinically important difference, at 18 months post randomization, of 1.5 on the EDE, with a pooled standard deviation of 1.4 [38]. The MBT arm consists of a group treatment so has been inflated to allow for correlation between the measures of those participants in the same group therapy Assuming an average group size of 10 and an intraclass correlation between Global EDE scores of 0.07 we considered a design effect of 1.63.

#### Analysis

Statistical analysis will be carried out in Stata. Mediation modeling may require the use of specialist structural equation modeling software (e.g. Mplus). All treatment group comparisons will be carried out on an intention-to-treat basis, that is subjects will be analysed in the group to which they were randomised irrespectively of the treatment received.

The primary and secondary longitudinal outcomes will be analyzed using linear mixed modelling (LLM) [51]. In this model the outcome variable measured at the post treatment time points (here 6, 12 and 18 months) is the dependent variable with outcome at baseline (EDE), treatment (MBT or SSCM), time (12 or 18 months), a treatment × time interaction and stratification factors (BMI and service type) as covariates. To account for correlation between repeated measures on the same individual a subject varying intercept will be included. To allow for correlation between EDE measures within the same therapy group, a further random effect will be included that varies with therapy arm.

Treatment effects on any secondary outcomes will be assessed in the same way, allowing for any deviations from a non-normal data by generalisations of the LMM.

Analyses that are based on maximum likelihood and will provide valid inferences under a missing at random (MAR) missingness mechanism. Sensitivity analyses will be used to assess the robustness of conclusions to missing outcome data and to departures from randomised treatment in the manner of [52].

Analyses of mediation at 18 months will use a regression approach as described by Holmbeck [53], [54], together with more recent statistical methods [54] to allow for possible hidden confounding between the mediator and outcome.

#### Economic evaluation

The economic evaluation will take a NHS/PSS perspective as recommended by NICE, including the cost of all hospital and community health and social care services. Resource use information will be collected using a modified version of the Adult Service Use Schedule (AD-SUS). Resource use data will be combined with appropriate national unit costs to calculate the total cost of the intervention and control groups. The cost of the MBT will be directly calculated from salaries using a micro-costing approach [55]. Differences in mean total costs between groups will be compared using the standard *t*-test with ordinary least squares regression used for adjusted analyses and the validity of results confirmed using bootstrapping [56]. The cost-effectiveness of the intervention will be assessed through the calculation of incremental cost effectiveness ratios, using the primary outcome measure and cost-utility will be assessed through the calculation of QALYs based on the EQ-5D. Uncertainty around the cost and effectiveness estimates will be represented using cost-effectiveness acceptability curves [57].

### Ethical review and trial registration

The study was reviewed by the South East Research Ethics Committee (Reference 10/H1102/2) and a favourable opinion was granted on 15/2/10.

Trial registration: The trial was registered on Current Controlled Trials ISRCTN51304415.

## Discussion

This innovative project will evaluate a modification of an established treatment in a patient population with a common and serious combination of conditions which are dangerous, sometimes fatal, and currently treated by multiple agencies which often fail adequately to meet their needs. Patients with symptoms of both BPD and Eating Disorder are treated by separate services addressing nutrition, drug abuse, self-harm and mood and impulse control and other problems. In this study, Mentalization Based Therapy which has been found effective in patients with BPD alone, will form the core of the treatment which will be administered by a single team, with the help of emergency services as needed and all participants will receive dietetic counselling. If effective, the treatment would be relevant to the care of many seriously ill, often young people who utilize substantial health care resources across several health sectors and who are currently regarded as untreatable by some clinical staff. Because of the efficacy of Mentalization Based Therapy in BPD, services offering MBT have been developed in many parts of the UK, and the project could encourage collaboration between such services and eating disorder units so that individuals with both Eating Disorder and BPD symptoms can be offered effective treatment.

Currently a patient with a combination of an eating disorder such as bulimia nervosa and a BPD may be referred to an eating disorders unit, a general psychiatric team for management of self-harm and depression, a psychotherapy service, a substance misuse service for help with cocaine or [58] alcohol abuse and may also present to the GP, or A and E for emergency treatment of suicidal or self-harming behaviour such as a drug overdose or self-cutting. There is no established treatment for this group, although both specialized inpatient care [59] and outpatient psychotherapy have been tried. If MBT-ED is found to be helpful, it could be provided wherever staff trained in outpatient delivery of the approach are available. Training for the treatment of BPD using Mentalization Based Therapy is already being provided on a fairly large scale, and the same could be achieved for the treatment of patients with both Eating Disorder and BPD using the newly developed MBT-ED. Existing training for MBT requires a short (3 day) course which, together with specialist MBT supervision allows staff to take on patients and to co-facilitate MBT groups in a clinical setting with usual NHS provision of resources. The patients are already being referred and seen by Mental Health Staff including nurses, psychologists and doctors, and these staff could be trained in the new approach and patients helped to avoid crisis care, including inpatient admissions. Existing staff time, which is currently not used effectively because of poor compliance and crises, would be redirected to a treatment approach that could lead to long term improvement and a reduced need for health care, currently provided in diverse and sometimes poorly co-ordinated services.

The Aetiologies of both Borderline Personality Disorder and Eating disorders are incompletely understood. It has been suggested [60] that experiences in infancy interacting with genetic and developmental processes, can contribute to deficits in mentalization which are found in these conditions, raising the possibility that there may be a contribution to their aetiology which is located during the first decade of life. This study, by investigating mentalization and object relations in participants undergoing different forms of therapy may provide useful information which bears on these ideas.

## Endnote

^a^The Reflective Function Questionnaire. Fonagy, P. 2010. Personal communication.

## Competing interests

The authors declare that they have no competing interests.

## Authors’ contributions

PR: Chief investigator, Principle investigator, St Ann’s Hospital. BB: Health economics design and analysis. AB: Trial design, model adherence for MBT. AH: Principle Investigator, Dartmouth Park Unit. JH: Trial statistician. FL: User consultant/expert by experience. CR: Statistics design. US, Principle Investigator, South London and the Maudsley Hospital. PF: Trial design, MBT theoretical aspects. All authors read and approved the final manuscript.

## Pre-publication history

The pre-publication history for this paper can be accessed here:

http://www.biomedcentral.com/1471-244X/14/51/prepub
